# Orexin A as a modulator of dorsal lateral geniculate neuronal activity: a comprehensive electrophysiological study on adult rats

**DOI:** 10.1038/s41598-019-53012-9

**Published:** 2019-11-13

**Authors:** Patrycja Orlowska-Feuer, Magdalena Kinga Smyk, Katarzyna Palus-Chramiec, Katarzyna Dyl, Marian Henryk Lewandowski

**Affiliations:** 1 Malopolska Centre of Biotechnology (MCB), Jagiellonian University in Krakow, Krakow, Poland; 2 Department of Neurophysiology and Chronobiology, Institute of Zoology and Biomedical Research, Jagiellonian University in Krakow, Krakow, Poland

**Keywords:** Orexin, Thalamus, Sensory processing

## Abstract

Orexins (OXA, OXB) are hypothalamic peptides playing crucial roles in arousal, feeding, social and reward-related behaviours. A recent study on juvenile rats suggested their involvement in vision modulation due to their direct action on dorsal lateral geniculate (dLGN) neurons. The present study aimed to verify whether a similar action of OXA can be observed in adulthood. Thus, *in vivo* and *in vitro* electrophysiological recordings on adult Wistar rats across light-dark and cortical cycles were conducted under urethane anaesthesia. OXA influenced ~28% of dLGN neurons recorded *in vivo* by either excitation or suppression of neuronal firing. OXA-responsive neurons did not show any spatial distribution nor represent a coherent group of dLGN cells, and responded to OXA similarly across the light–dark cycle. Interestingly, some OXA-responsive neurons worked in a cortical state-dependent manner, especially during the dark phase, and ‘preferred’ cortical activation over slow-wave activity induced by urethane. The corresponding patch clamp study confirmed these results by showing that < 20% of dLGN neurons were excited by OXA under both light regimes. The results suggest that OXA is involved in the development of the visual system rather than in visual processes and further implicate OXA in the mediation of circadian and arousal-related activity.

## Introduction

Orexin A and B (OXA and OXB), also known as hypocretin 1 and 2, are hypothalamic neuropeptides^1,2^ involved in diverse physiological functions such as feeding, regulation of the sleep-wake cycle, homeostasis, stress responses and motivation^2–6^. Derived from a common precursor prepro-orexin and synthetized exclusively in the lateral hypothalamus (LH), orexins bind to two G protein-coupled receptors: OX_1_ selective for OXA and non-selective OX_2_, binding both peptides with similar affinity^2^. Orexins exert their multiple effects by means of extensive projections throughout the brain^1,7^.

Engaged in the regulation of rhythmically occurring processes (e.g., sleep-wake cycle) and controlled by a master circadian pacemaker the suprachiasmatic nucleus (SCN)^8–11^, orexins follow a circadian pattern of expression and action. Significant daily variation has been found for the level of prepro-orexin mRNA and OXA immunoreactivity in the hypothalamus^12–14^, OXA in the cerebrospinal fluid^9,10,15^, as well as the number and activity of orexin neurons in rodents^16,17^. Increased levels of peptide production, expression and associated neuronal activity have been observed during the dark phase of the photoperiod, which in nocturnal rodents is a time of prolonged wakefulness and motor activity^10,15,16^. Indeed, orexin neurons increase their firing rate during active wakefulness, decrease the frequency of discharges when animals approach sleep and become silent during slow-wave and REM sleep^18,19^. Considered as a neuroexcitatory agent^1,20–23^, OXA has also been found to inhibit neuronal activity^24,25^. Interestingly, day/night differences have been reported for this mechanism of action in the SCN^26^.

Recently, an *in vitro* study showed that OXA exerts multiple excitatory actions on neurons in the dorsal lateral geniculate nucleus (dLGN), a thalamic relay for vision^27^. Therefore, this sensory modality has been suggested to be modulated by arousal and a circadian factor, both of which are related to orexins^27^. Certainly, visual processing in the dLGN differs according to vigilance state^28,29^, and the neuronal activity of the dLGN has recently been reported to be influenced by the general state of the brain under different types of anaesthesia^30,31^.

The present study aimed to investigate the putative action of OXA on neuronal activity in the dLGN in adult Wistar rats *in vivo* and *in vitro*. Considering the diurnal variation in the actions of orexins, as well as orexinergic fibre density in the dLGN^27^, the peptide was applied in both phases of the photoperiod. Additionally, the light responsiveness, relationship with the general brain state, regularity of the discharge pattern and presence of infra-slow oscillatory activity of dLGN neurons were characterised.

## Results

**Figure 1:**
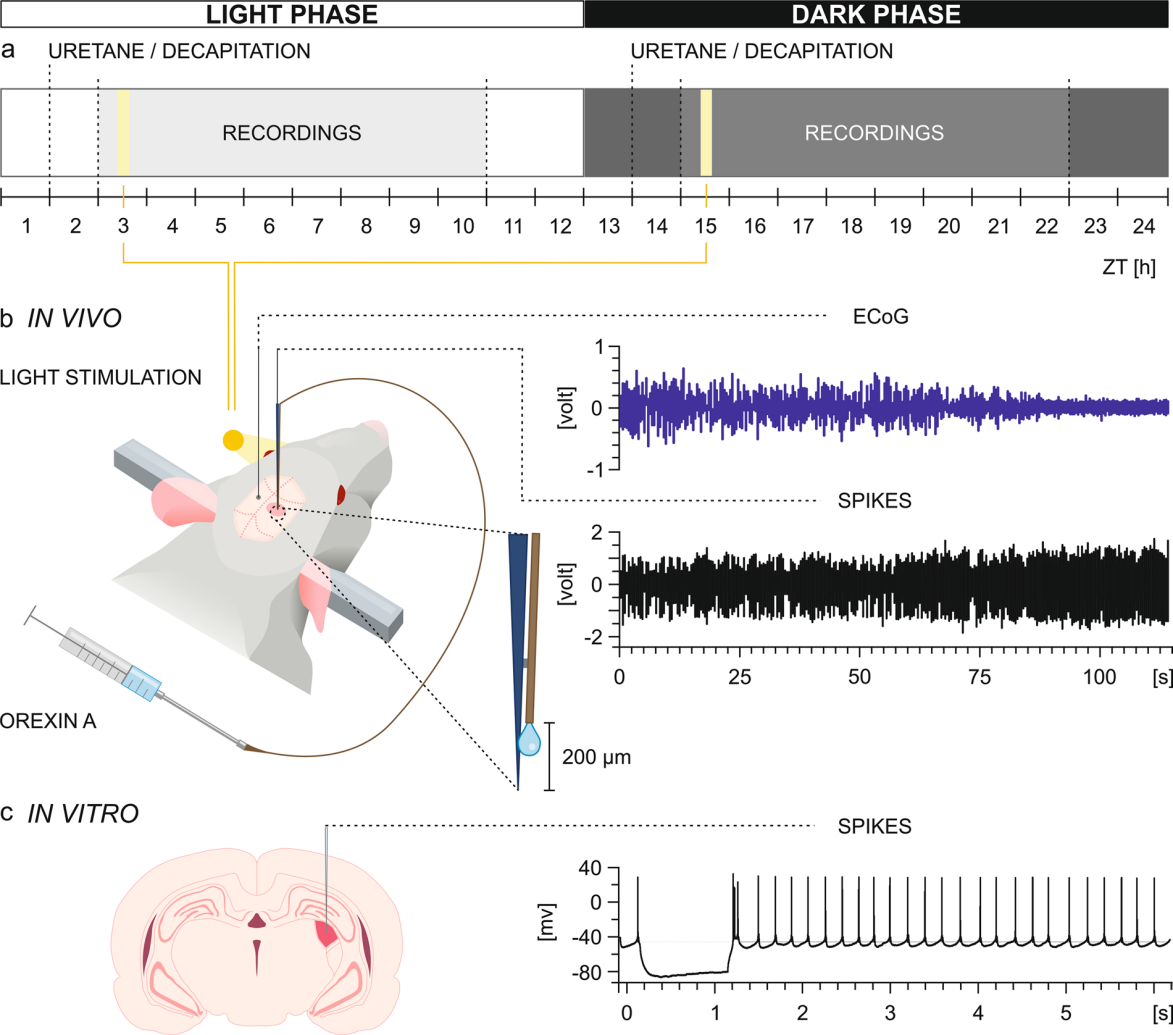
Experimental design. (**a**) The plan depicting the lighting conditions under which experiments were conducted. (**b**) A schematic drawing of the *in vivo* experimental design with examples of simultaneously recorded raw ECoG and neuronal activity signals and magnification of the recording electrode connected to the custom-made injection system enabling local, pressure-driven OXA infusions. (**c**) A schematic drawing of the *in vitro* experimental design with an example of a raw signal recorded from the dLGN (coloured red on the coronal slice).

The recent *patch clamp* study on juvenile Wistar rats suggested orexin involvement in vision modulation due to the direct, excitatory action of orexins on dLGN neurons. Moreover, immunohistochemical staining showed sparse, circadially modulated orexinergic innervation in the dLGN^27^. We aimed to verify whether similar action of OXA can be observed in adulthood and, if so, whether it is influenced by the time of day. Therefore, we compared sensitivity of dLGN neurons to OXA application during *in vivo* and *in vitro* recordings under two light regimes: light and dark (Fig. 1). The dLGN is known as the main thalamic relay centre for the visual pathway due to direct retinal innervation. Thus, the majority of recorded neurons were tested for light responsiveness. Quite recently it has been showed that neuronal activity within the dLGN is modulated by the general brain state^30,31^, accordingly, we aimed to describe OXA-responsive neurons also regarding this particular property. A subpopulation of dLGN cells was previously found to express infra-slow oscillatory activity^32–34^; hence, we verified whether such neurons are influenced by OXA.

**Figure 2:**
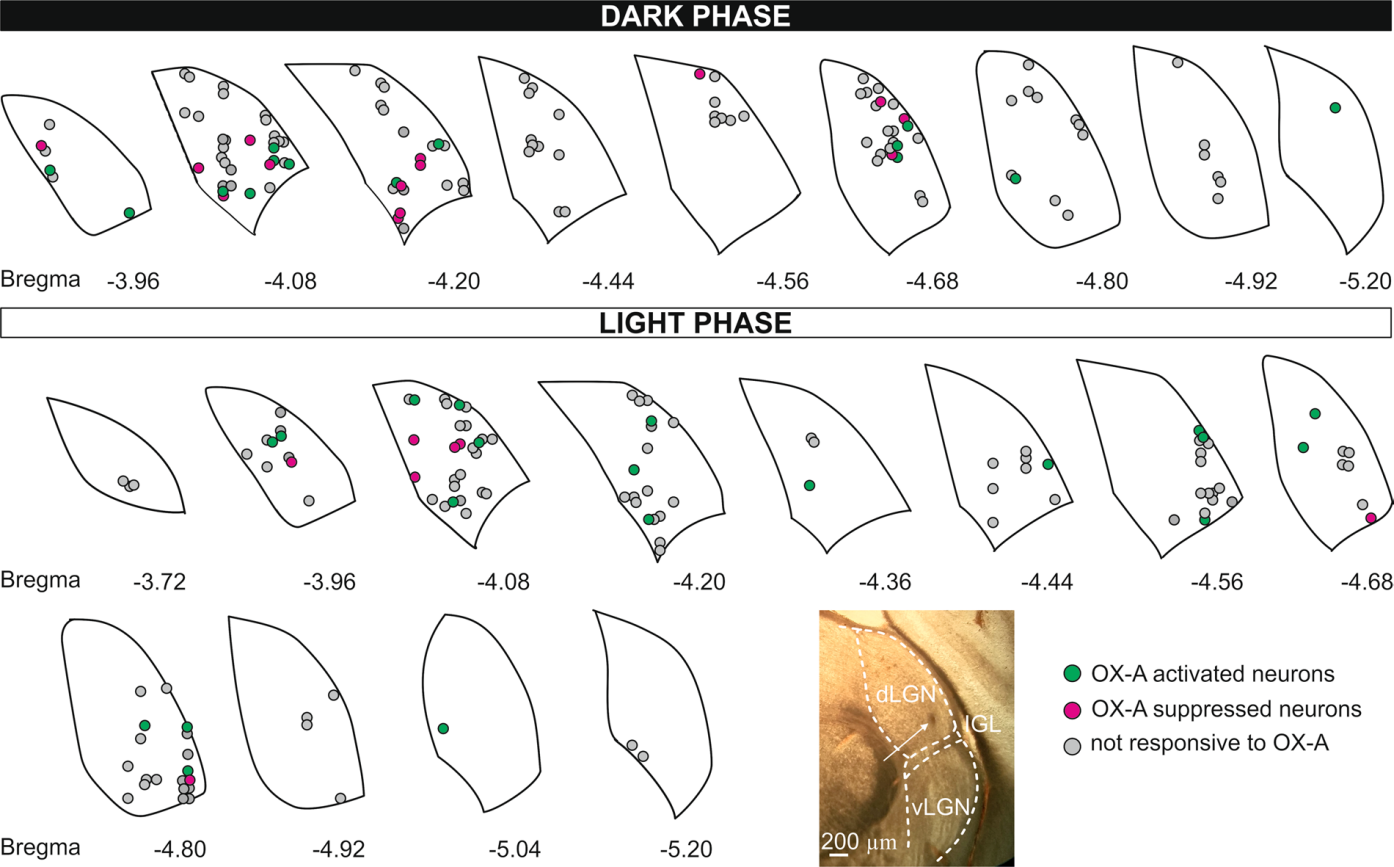
Localisation of *in vivo* recording sites. Estimated anatomical locations (based on the ChSB marks visualised under the microscope as shown on a representative image) of all recorded dLGN cells under the dark and light phase plotted on coronal diagrams (various distance from *Bregma*) from the stereotaxic rat brain atlas^100^. OXA-responsive neurons (colour-coded) in the dLGN were distributed across the structure. Dashed curves on the image outline the borders of the nuclei: dLGN – dorsal lateral geniculate nucleus, IGL – intergeniculate leaflet, vLGN – ventral latera geniculate nucleus.

In total, the activity of 235 dLGN neurons was recorded *in vivo* under urethane anaesthesia: 118 under photopic conditions during the light phase (100 µW/cm^2^, ZT 3–10; 25 rats) and 117 under scotopic conditions during the dark phase (>0.1 µW/cm^2^, ZT 15–22; 19 rats). The recorded neurons were evenly distributed across the entire dLGN, as shown in Fig. 2. Detailed electrophysiological characterization of all recorded neurons is presented in Supplementary Table S1, Figs S1, S2.

### Orexin A influences the spontaneous firing rate of dLGN neurons *in vivo* during the light phase

**Figure 3:**
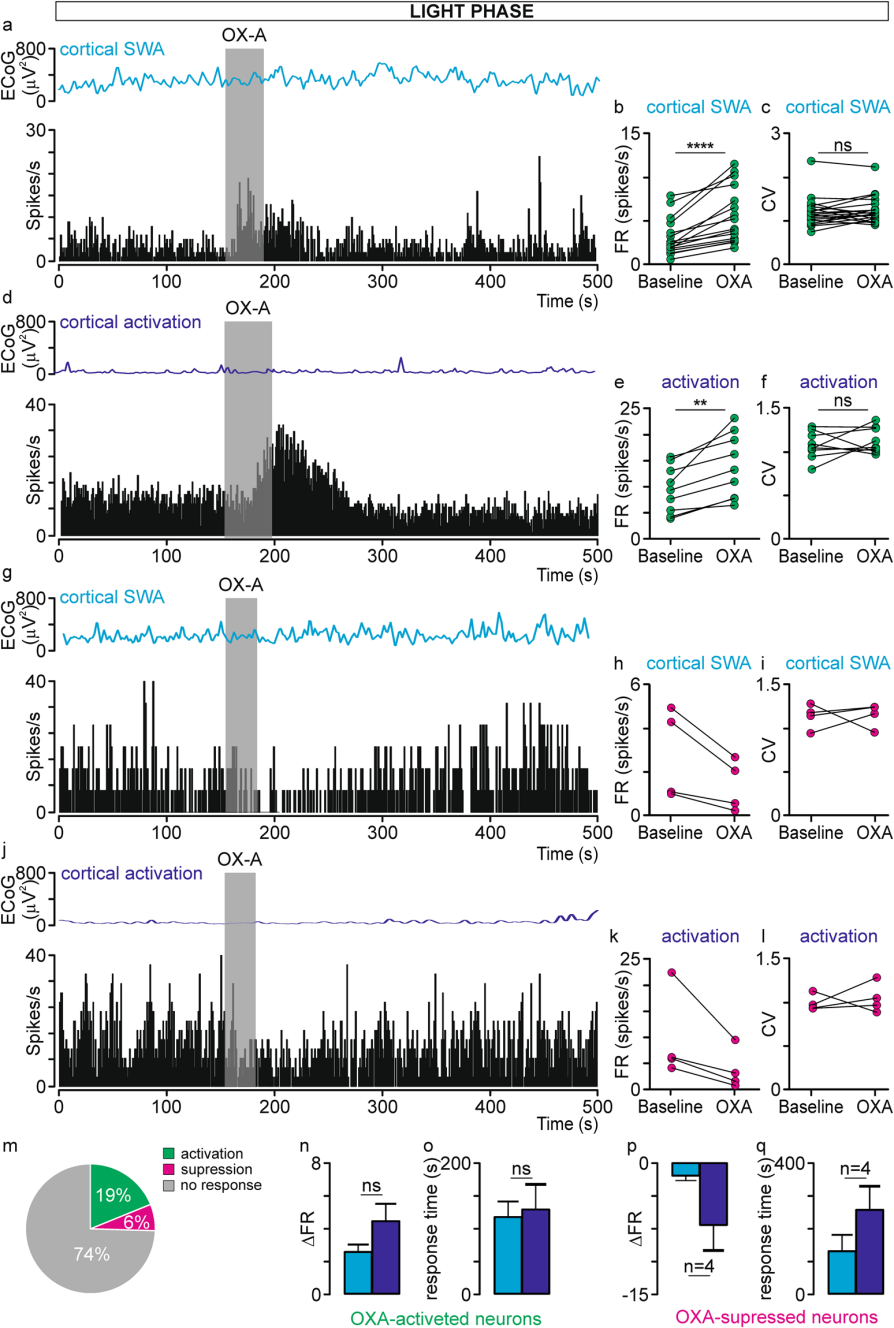
OXA influences spontaneous neuronal activity in the rat dLGN *in vivo* under the light phase. Representative traces of simultaneously recorded OXA-responsive dLGN neurons and ECoG presented as a delta-band ECoG power during (**a, g**) cortical SWA and (**d, j**) activation phases and adequate firing rate histograms of dLGN neurons (bin size = 1 s). Grey rectangles denote the time of OXA infusions. The mean ± SEM of OXA-activated neurons (whose firing rate was increased upon OXA infusion) recorded during cortical (**b**) SWA and (**e**) activation phases. The mean ± SEM coefficient of variation (CV) for ISI histograms were compared between neuronal activity before and after OXA infusion for OXA-activated neurons recorded during cortical (**c**) SWA and (**f**) activation phases. The mean ± SEM of OXA-supressed neurons (whose firing rate was decreased upon OXA infusion) recorded during cortical (**h**) SWA and (**k**) activation phases. The mean ± SEM coefficient of variation (CV) for ISI histograms were compared between neuronal activity before and after OXA infusion for OXA-supressed neurons recorded during cortical (**i**) SWA and (**l**) activation phases. (**m**) Summary of population data showing the proportion of OXA-responsive dLGN neurons recorded *in vivo* under the light phase (n = 106). (**n, p**) The change in firing rate (∆FR) ± SEM upon OXA application was compared between cortical states to verify whether the magnitude of response is modulated by general brain alterations. (**o, q**) The response time was also compared between OXA-responsive neurons and across cortical state. Data in (**b,c,e,f,o,p and q**) were analysed by Wilcoxon matched-pairs signed rank test and paired t-test, and data in (**n)** by Mann-Whitney test; ** p < 0.01, **** p < 0.0001, ns > 0.05.

The effect of 200 µM OXA infusion on the spontaneous firing of dLGN neurons was first tested during the light phase. In total, 118 neurons were subjected to that experimental protocol; however, due to observed changes in the cortical state influencing the spontaneous firing rate of dLGN neurons during or just after OXA infusion (Fig. S1), only 106 neurons could be reliably analysed (100-s stabile baseline and 300-s post-infusion activity were compared). In 27 out of 106 dLGN cells, statistically significant changes (>3 SDs) in the firing rate were observed after pressure-driven OXA infusion, and both activation (n = 20) and suppression (n = 7) of firing were observed (Fig. 3). OXA-responsive neurons were further divided with respect to the cortical state during which they were tested; thus, four groups were characterised: activated (n = 16) and suppressed (n = 4) during slow-wave activity (SWA) and activated (n = 9) and suppressed (n = 4) during cortical activation. Importantly, Fisher’s exact test showed that there were no differences in the proportion of neurons responsive to OXA between cortical phases (p = 1.00). OXA increased the spontaneous firing of dLGN neurons from 3.09 ± 0.53 Hz to 5.67 ± 0.81 Hz (Wilcoxon matched-pairs signed rank test, p < 0.0001, W = 136.0; Fig. 3a,b) and 9.49 ± 1.51 Hz to 13.96 ± 2.06 Hz (paired t-test two-tailed, p = 0.0031, t = 4.172, df = 8; Fig. 3d,e) during SWA and cortical activation, respectively. The change in firing rate (∆FR) upon OXA infusion was compared between cortical states to verify whether the magnitude of response is modulated by cortical alterations. A slightly higher ∆FR was observed during cortical activation (Mann-Whitney test, two-tailed, p = 0.0953, U = 42.00; Fig. 3n). The mean response time upon OXA infusion did not differ between general brain states and was 118 ± 24 s and 130 ± 39 s during SWA and cortical activation, respectively (OXA-activated neurons were compared: Wilcoxon matched-pairs signed rank test, p = 0.6523, W = −9.0; Fig. 3o). Surprisingly, a small subset of neurons was suppressed by OXA application during the SWA (n = 4; Fig. 3g,h) and cortical activation (n = 4; Fig. 3j,k) states. Similarly as in the case of OXA-activated neurons, a higher ∆FR and longer response time were observed during cortical activation state, however the number of OXA-supressed neurons was too small to perform statistical comparison (Fig. 3p–r).

Importantly, 13 out of 27 OXA-responsive neurons were tested under both cortical states, and five different response types were observed: activation (n = 5) or suppression (n = 1) under both phases, activation (n = 3) or suppression (n = 3) only during cortical activation and activation (n = 1) only under SWA state, suggesting that OXA at least to some extent works in a state-dependent manner and that alterations in the ECoG may influence dLGN neuron physiology, as the responses were often observed only during one cortical phase.

All neurons responsive to OXA (either activated or supressed) were characterised as non-regular with a mean CV of 1.20 ± 0.08 and 1.02 ± 0.04 during the SWA and cortical activation state, respectively, and their CV did not differ from the mean CV of neurons not responsive to OXA (unpaired t-test, two-tailed; SWA: p = 0.3376, t = 0.9561, df = 74; activation state: p = 0.3834, t = 0.8787, df = 55). Moreover, the mean CV was not changed during the response to OXA (SWA: Wilcoxon matched-pairs signed rank test, two-tailed, p = 0.3488, W = 52.00, n = 20; activation state: Paired t-test, two-tailed, p = 0.3485, t = 0.9757, df = 12, n = 13; Fig. 3c, f).

Electrophysiological characteristics of OXA-responsive dLGN neurons showed that 19 out of 23 tested neurons were modulated by the cortical state (4 were only recorded during one cortical phase), 12 out of 26 tested were sensitive to light stimulation (light was presented mostly during SWA phase, however sometimes such verification was also performed during cortical activation state) and 8 exhibited both of these properties. Moreover, infra-slow oscillatory neurons (n = 3) were among these cells. Among OXA-responsive neurons receiving retinal input, all three known types^35^ of light responses were observed: transient ON (characterised by a peak of activity at the light onset and sometimes offset), OFF (exhibited a rebound activation after the stimulus termination) and sustained (maintained elevated firing during the stimulus presentation). The light-induced responses were assessed based on the peristimulus time histograms (bin size = 0.1 s) and 6 OXA-responsive neurons were characterised as transient ON, 4 as transient OFF and 1 as sustained. Among OXA-activated neurons were transient ON, transient OFF, sustained and non-responsive types, while only 1 out of 7 OXA-supressed neurons was light responsive (transient OFF). Also, neurons responding to OXA application only during one cortical state could be characterised as transient ON, OFF or not light-responsive. The above analysis suggests that OXA-responsive dLGN neurons represent different neuronal populations, and only some of them (46%) are directly engaged in vision formation.

### Background lighting does not influence dLGN cell responsiveness to OXA during the light phase

The dLGN receives strong excitatory input from retinal ganglion cells, which significantly influences its spontaneous activity^36^. Thus, we decided to verify whether a light background impacts the ability of dLGN cells to respond to OXA infusion. To this end, electrophysiological recordings were performed during the light phase under a photopic and scotopic lighting background. In total, 22 neurons were subjected to that protocol, and the response of each of them to OXA was tested four times (infusions during both cortical states under a photopic and scotopic background). OXA infusion statistically (≥3 SD) influenced the activity of 8 dLGN neurons, and both activation (n = 4) and suppression (n = 4) of firing were observed. Among OXA-responsive cells, 3 responded under all four conditions tested (2 cells were activated and 1 suppressed), and 5 responded only during the cortical activation state (2 cells were activated and 3 were suppressed). Importantly, there were no changes in cells responsiveness to OXA between background lighting conditions, meaning that if cells responded to the drug under scotopic conditions, they also responded under photopic conditions. In this way, we ruled out the possibility that a high firing rate of dLGN neurons under photopic conditions makes neurons insusceptible to OXA. Moreover, these observations confirm the above result suggesting that OXA may work in a state-dependent manner in the dLGN. Due to the small sample size, no statistical test could be performed; however, stronger suppression of firing was observed under a photopic background, most likely due to the higher spontaneous firing rate (activation state: scotopic conditions, ∆FR: −7.15 ± 2.85 Hz, photopic conditions, ∆FR: −2.54 ± 0.95 Hz).

### Orexin A influences the spontaneous firing rate of dLGN neurons *in vivo* during the dark phase

**Figure 4:**
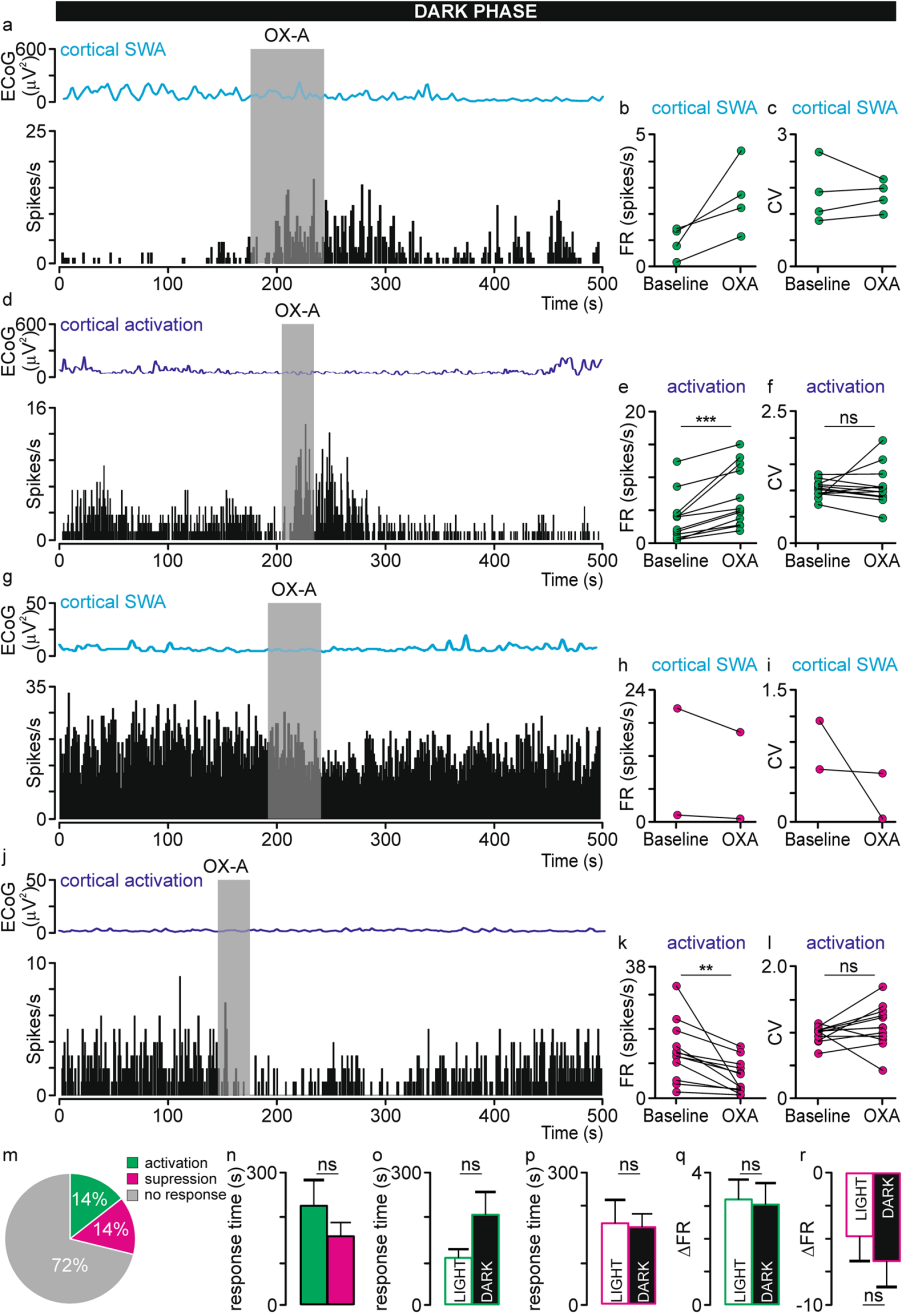
OXA influences spontaneous neuronal activity in the rat dLGN *in vivo* under the dark phase. Representative traces of simultaneously recorded OXA-responsive dLGN neurons and ECoG presented as a delta-band ECoG power during (**a, g**) cortical SWA and (**d, j**) activation phases and adequate firing rate histograms of dLGN neurons (bin size = 1 s). Grey rectangles denote the time of OXA infusions. The mean ± SEM of OXA-activated neurons (whose firing rate was increased upon OXA infusion) recorded during cortical (**b**) SWA and (**e**) activation phases. The mean ± SEM coefficient of variation (CV) for ISI histograms were compared between neuronal activity before and after OXA infusion for OXA-activated neurons recorded during cortical (**c**) SWA and (**f**) activation phases. The mean ± SEM of OXA-supressed neurons (whose firing rate was decreased upon OXA infusion) recorded during cortical (**h**) SWA and (**k**) activation phases. The mean ± SEM coefficient of variation (CV) for ISI histograms were compared between neuronal activity before and after OXA infusion for OXA-supressed neurons recorded during cortical (**i**) SWA and (**l**) activation phases. (**m**) Summary of population data showing the proportion of OXA-responsive dLGN neurons recorded *in vivo* under the dark phase (n = 97). (**n**) The response time did not differ between activated and supressed neurons recorded during cortical activation state. Also, the response time did not differ between (**o**) activated and (**p**) supressed neurons recorded during light and dark phases. (**q, r**) The change in firing rate (∆FR) ± SEM upon OXA application was compared between lightening regimes to verify whether the magnitude of response is influenced by circadian time. Data in (**e, k, l**) were analysed by Wilcoxon matched-pairs signed rank test and paired t-test, and data in (**n–r**) by Mann-Whitney test and unpaired t-test; ** p < 0.01, *** p < 0.001, ns > 0.05.

Next, we set out to verify whether stronger orexinergic innervation of the dLGN during the dark phase is reflected in the electrophysiological sensitivity of these neurons to OXA infusion^27^. Thus, the effect of 200 µM OXA pressure-driven infusion on the spontaneous firing of dLGN neurons was tested during the dark phase. In total, 117 cells were subjected to that experimental protocol; however, only 97 were further analysed. OXA infusion significantly influenced the spontaneous firing rate of 28% of tested dLGN neurons, and both activation (n = 14) and suppression (n = 14) of activity were observed (Fig. 4). The proportion of OXA-responsive neurons recorded during the lightening regimes did not differ (Fisher’s exact test, p = 0.6368); however, suppression of activity upon OXA infusion was observed slightly more often during the dark phase (Fisher’s exact test, p = 0.0966). OXA-responsive dLGN neurons were further divided with respect to the cortical phase during which they were recorded, and four groups were characterised: activated (n = 4) or suppressed (n = 2) during SWA and activated (n = 12) or suppressed (n = 12) during cortical activation (Fig. 4). In contrast to the observations in the light phase, Fisher’s exact test showed that OXA-responsive neurons were more often found during the cortical activation brain state. OXA infusion significantly increased dLGN firing from 3.35 ± 1.06 Hz to 6.72 ± 1.36 Hz (Wilcoxon matched-pairs signed rank test, p = 0.0005, W = 78.00, n = 12, Fig. 4d,e) or decreased it from 13.39 ± 2.47 Hz to 6.01 ± 1.39 Hz (paired t-test two-tailed, p = 0.0074, t = 3.271; df = 11, n = 12, Fig. 4j,k). The mean response time did not differ between activated and suppressed neurons recorded under the cortical activation state and lasted 225 ± 59 s and 156 ± 32 s, respectively (Mann-Whitney test, p = 0.5041, U = 60.00, Fig. 4n). Importantly, 7 out of 28 OXA-responsive neurons were tested during both cortical states, and 2 of them were activated during both brain states, whereas the remaining 5 were influenced only during the cortical activation state (2 were activated and 3 were suppressed), further confirming that some OXA-responsive dLGN neurons work in a state-dependent manner.

The mean CV of OXA-responsive neurons was 1.23 ± 0.08 and 1.00 ± 0.02 during cortical SWA and activation states, respectively, and did not differ from that of neurons not responsive to OXA. Furthermore, OXA application did not influence the regularity of spiking in responding dLGN neurons (activation during cortical activation: paired t-test, two-tailed, p = 0.5988, t = 0.5418, df = 11, n = 12; suppression during cortical activation: Wilcoxon matched-pairs signed rank test, p = 0.1294, W = 40.00, n = 12; Fig. 4f, l).

Electrophysiologically, OXA-responsive neurons in the dark phase were, in the majority, modulated by the general brain state (20/22 tested) and received retinal innervation (17/28 from which 11 were characterised during SWA phase and exhibited transient ON (n = 1), OFF (n = 3) and sustained (n = 6) responses). Moreover, 11 of them expressed both of these properties. Similarly like in the light phase, responses to light could not be linked to any particular type of OXA-responding neurons, as transient ON, OFF and sustained responses were observed in activated and supressed neurons.

### Comparison between OXA-responsive dLGN neurons recorded *in vivo* under the light and dark period

Finally, we compared the electrophysiological properties of OXA-responsive dLGN neurons recorded during the light and dark periods. There were no differences in the magnitude of the response (calculated as the average change in ∆FR) and the mean time of the response between OXA-activated neurons (the response time: Mann-Whitney test, p = 0.0522, U = 84.5, Fig. 4o; magnitude of the response: Mann-Whitney test, p = 0.8911, U = 136.00, Fig. 4q) and OXA-supressed neurons (the response time: unpaired t-test two-tailed, p = 0.8779, t = 0.1557, df = 19; Fig. 4p; magnitude of the response: Mann-Whitney test, p = 0.4862, U = 39.00, Fig. 4r) recorded *in vivo*. Interestingly, significantly more neurons were influenced by OXA during cortical activation state in the dark phase than in the light phase (Fisher’s exact test, p < 0.0001).

### Lack of circadian modulation in responsiveness to OXA of dLGN neurons recorded *in vitro*

Next, we turned our attention towards patch clamp recordings from dLGN slices obtained from adult rats to verify whether the differences observed between the presented results and Chrobok *et al*.^27^ were due to developmental changes of the visual system, as the previous work was performed on juvenile Wistar rats.

**Figure 5:**
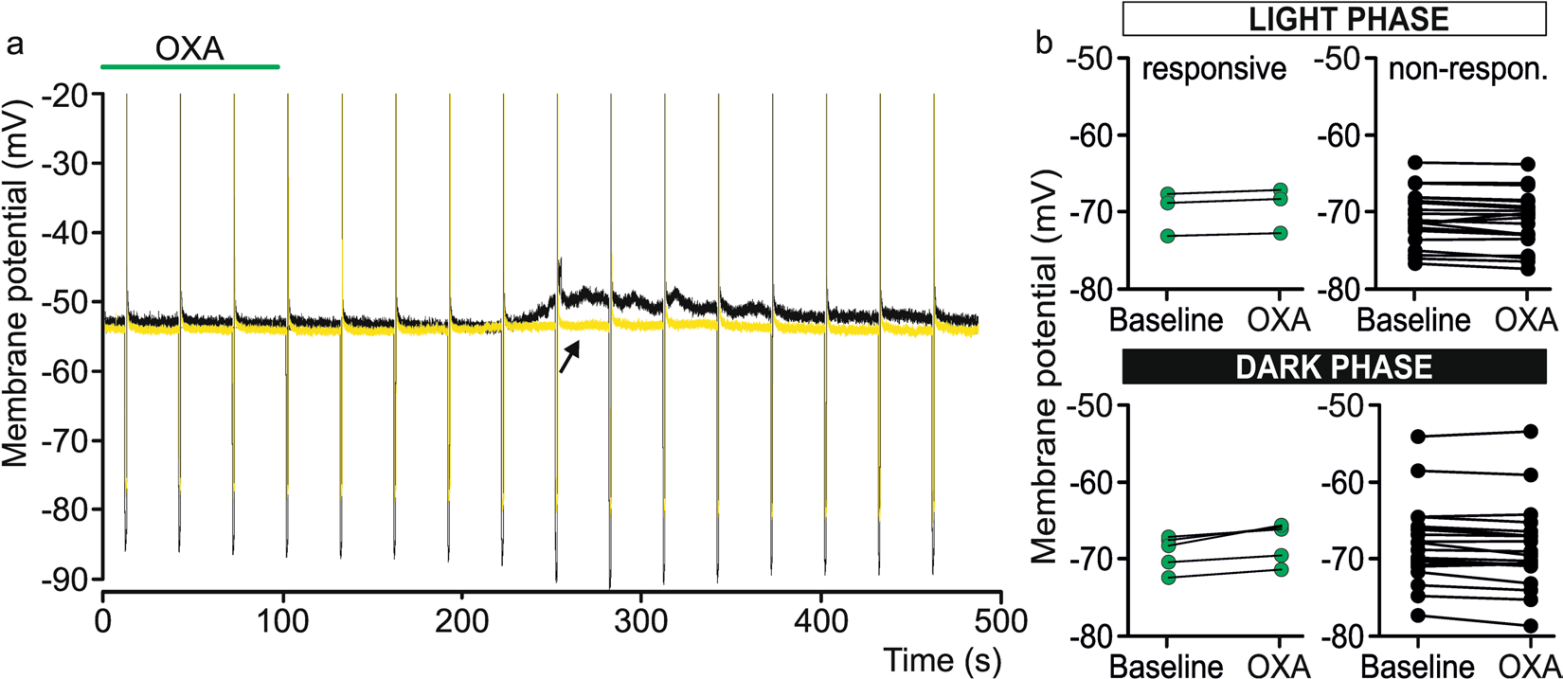
Lack of circadian modulation in responsiveness to OXA of dLGN neurons recorded *in vitro*. Representative patch clamp recordings from dLGN neurons (yellow trace – recording from light phase, black trace – recording from dark phase) and their depolarizing responses to OXA (200 nM, green bar) application. Deflections represent voltage responses to rectangular pulses of current (80 pA). (**b**) The effect of OXA (200 nM) on the membrane potential of the dLGN neurons in ACSF in both phases (light and dark, respectively). Black dots and lines indicate neurons that were insensitive to peptide administration (non-responsive), and green dots and lines represent dLGN cells that were depolarised by OXA (responsive).

In total, 50 neurons were recorded (25 neurons per phase). In both phases, responses to OXA application were observed, but were much more rare (8/50) than in juvenile rats^27^. During the light period, only 12% of recorded neurons were sensitive to OXA application, causing rather small depolarisation (Δ_max_: 0.60 ± 0.03 mV, n = 3/25, Fig. 5). In the dark period, 20% of recorded neurons were affected by OXA application, and a tendency for higher depolarisation was observed (Δ_max_: 2.03 ± 0.55 mV, n = 5/25, Fig. 5). Similar to *in vivo* preparations, there were no significant differences between the proportion of OXA-responsive dLGN neurons recorded during the light and dark period (Fisher’s exact test, p = 0.7019).

### Sparse orexinergic innervation of the dLGN in juvenile rats

**Figure 6:**
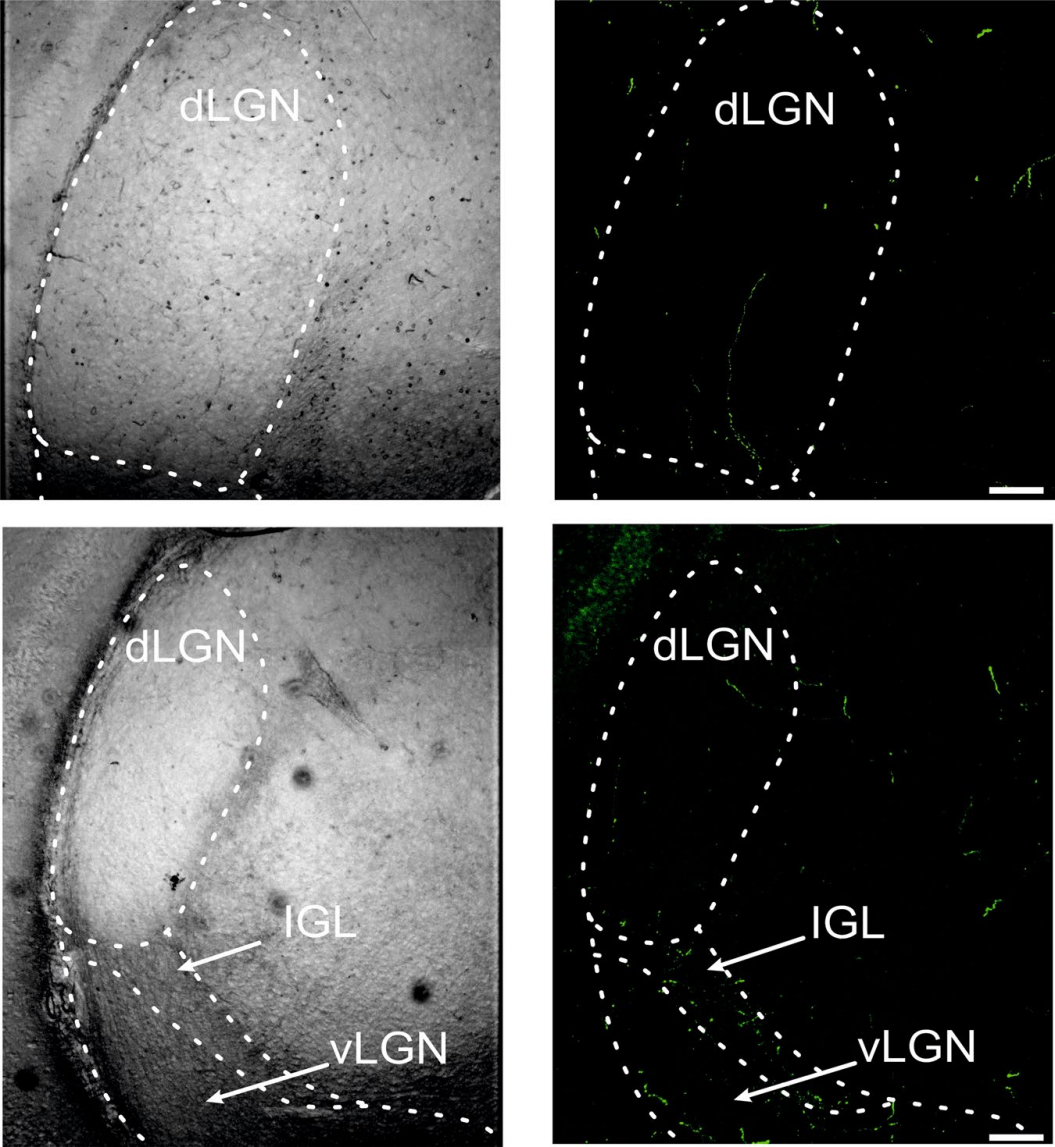
Sparse orexinergic innervation of the dLGN in juvenile rats. A representative OXA immunofluorescent labelling of juvenile Wistar rat dLGN (green, right panel). Left panel represents pictures under bright light. The white bars represent 100 μm. Dashed curves on the images outline the borders of the nuclei: dLGN – dorsal lateral geniculate nucleus, IGL – intergeniculate leaflet and vLGN – ventral lateral geniculate nucleus.

To verify whether the discrepancies between the presented results and Chrobok *et al*.^27^ were due to developmental changes in orexinergic innervation of the dLGN, an additional immunohistochemical experiment was performed. Using the same technique as Chrobok *et al*.^27^ we showed that the dLGN of juvenile rats is sparsely innervated by orexinergic fibres (mean area fraction: 0.096 ± 0.017; Fig. 6).

## Discussion

To our knowledge, this is the first study showing that OXA causes long-lasting effects (excitation or inhibition; 2–3 min long) in ~28% of dLGN neurons recorded *in vivo* under the light and dark phase, thus when neurons receive multiple neuronal inputs according to the Zeitgeber Time. OXA-responsive neurons did not show any spatial distribution and did not represent any coherent electrophysiological group of dLGN cells. Moreover, dLGN neurons responded to OXA similarly during the day and the night, as there were no differences in the magnitude of the response or the mean response time to OXA between light regimes. Interestingly, the present results show that OXA-responsive dLGN neurons can work in a state-dependent manner, especially during the dark phase, and ‘prefer’ cortical activation rather than the SWA state induced by urethane anaesthesia. Patch clamp recordings confirmed the results of the *in vivo* experiments and showed that <20% of dLGN neurons are excited by OXA under both light regimes. The magnitude of the response was slightly higher during the dark period, whereas the mean response time did not differ between phases and lasted ~3 min.

The effects of orexins on dLGN activity were studied before in slice preparation by three different groups^27,37,38^, and some inconsistencies were found. Reports by Bayer *et al*.^23^ and Govindaiah & Cox^38^ did not find any orexin-responsive neurons in the dLGN; however, they performed an overview of orexin action on different thalamic neurons. The total number of tested neurons in the dLGN in both studies was relatively low thus, orexin-responsive neurons may have been omitted from the recorded populations. On the other hand, Chrobok *et al*.^27^ focused on orexin action on the rat dLGN by performing a comprehensive study on Wistar rats. The authors showed that ~75% of dLGN neurons are excited by orexins regardless of light regime and with the involvement of voltage-dependent calcium influx and closure of GIRK channels. Moreover, their immunohistochemical study revealed sparse orexinergic innervation of the dLGN with maximal fibre density during the dark period. The discrepancies between the high number of orexin-responsive neurons and the sparse orexinergic innervation of the dLGN were explained by a possible engagement of volume transmission. Interestingly, however, we hypothesised that this difference may be due to developmental changes, as they used juvenile (14–21 days old) and adult (5–6 weeks old) rats for electrophysiology and immunohistochemistry, respectively. Rodent retina and the visual system are developed at the time of eye-opening (P12–P14); however, the functionality of the retina and specificity of the retinogeniculate synapse take approximately three weeks spanning eye opening to display the activity and connectivity typical of adult animals^39–43^. In fact, the present study confirms this hypothesis by showing that a relatively lower proportion of dLGN neurons are sensitive to OXA in *in vitro* (~20%) and *in vivo* (~28%) preparations in adult rats. Notably, we used the same technique (patch clamp recordings) as Chrobok *et al*.^27^ to determine the effects of OXA on dLGN neurons in adult rats; therefore, the observed difference between the results is not due to technical issues. Thus, the next obvious question is whether the dLGN of juvenile rats is densely innervated by orexinergic fibres. Surprisingly, our immunohistochemical data showed sparse orexinergic innervation of the dLGN in immature rats at ZT 1–2. Moreover, the orexinergic innervation did not follow any spatial pattern explaining random distribution of OXA-responsive neurons in the dLGN in our *in vivo* study. In the work of Chrobok *et al*.^27^ the density of orexinergic fibres was slightly higher in the anterior direction, however we did not observed such pattern. The quantitative analysis showed that in the present work the mean area fraction of orexinergic fibres is a bit higher than in a previous work on adult animals. There are few explanations for that. First of all, confocal laser parameters during scanning are adjusted to the observed immunoreactivity, so some differences could appear there. Secondly, different parameters could be used in Bernsen’s adaptive thresholding method. Importantly, all previous studies examining orexinergic innervation of the brain showed dense orexinergic fibres in a wide range of brain areas but no or very low density in the dLGN^44–46^. The possible explanation for the discrepancies between sparse orexinergic innervation (the present data) and high sensitivity to OXs^27^ in the juvenile rat dLGN might be due to developmental changes in orexin receptors. In fact, remarkable differences in the mRNA levels of prepro-orexin and orexin receptors occur during the first weeks of the postnatal period. Moreover, developmental changes are specific for brain regions. The mRNA level of prepro-orexin in the LH^47,48^, OXR_1_ in the ventromedial hypothalamic area and OXR_2_ in the paraventricular nucleus^47^ increases until the third postnatal week and then stabilises to further decrease with age. The hypothalamic level of mRNA for OXR_1_ and OXR_2_ has been shown to decrease with brain maturity, similar to the cortical level of OXR_2_^49,50^. Moreover, OXR_1_ mRNA was present in the infant cerebellum but not detectable at all in maturity^49^. Similarly, expression of OXR_2_ mRNA in hypoglossal motoneurons peaks around P20 and then strongly decreases^51^. This difference in expression across development is not an unusual situation, as different peptide receptors, including those for vasopressin^52^ and somatostatin^53^, which are also present in the visual system^54^, have been found to be more strongly expressed in the developing than in the adult brain. As mentioned before, the dLGN changes remarkably during the first few weeks of the postnatal period, including changes in total RNA and DNA contents, which stabilises around P40^55^. Taking these rationales into account, we suggest that orexins may strongly influence dLGN postnatal development. Indeed, such a role for orexins in different brain regions has been suggested previously^56^. Alternatively, the low number of OXA-responsive neurons in the dLGN of adult animals may be connected to concentration-dependent activity of OXA^57,58^. In the present work concentrations of 200 nm and 200 µm were used for patch clamp and *in vivo* studies, respectively. Even though, similar results were acquired for both preparations, there is still a possibility that higher concentrations would have more broaden responses. On the other hand, generally OXs concentration in nanomolar and micromolar range was used for the *in vitro*^27,59–61^ and *in vivo*^62–64^ studies, respectively.

The present work shows that dLGN neurons may be excited or suppressed by OXA during *in vivo* recordings under urethane anaesthesia regardless of light regime. Importantly, urethane has already been proven to be a good anaesthetic for experiments investigating diurnal variations in electrophysiological recordings in the SCN^65–67^ and medial habenula^68^. Additionally, urethane induces cyclic fluctuations in cortical and/or brainstem activity mimicking sleep episodes: slow-wave and desynchronization states; thus, urethane anaesthesia was used previously as a model of sleep (but not sleep-wake cycle)^69–73^. OXs are generally considered excitatory in both *in vivo* and *in vitro* preparations. Excitation of spontaneous neuronal activity by OXs has been observed under urethane anaesthesia in the hippocampus^62^, globus pallidus^74^, substantia nigra pars compacta^63^, dorsal raphe (DRN)^75^ and laterodorsal tegmental nucleus (LTD)^64^. Moreover, increases in neuronal activity *in vitro* upon OX application have been showed in numerous brain structures, such as the locus coeruleus^76,77^, tuberomammillary nucleus^78^, DRN^79^, intergeniculate leaflet^59,80^ and paraventricular nucleus of the thalamus^61^. However, an inhibitory action of OXA is not rare. OXA mainly suppresses neuronal firing in the SCN^26,60,81,82^ with different mechanisms across the light-dark cycle^26^. Moreover, OXA inhibits the activity of neurons expressing melanin-concentrating hormone in the LH by acting on local GABAergic interneurons^83^ and *OX*_1_*R* mRNA expression was found on the putative GABAergic interneurons in the LTD and pedunculopontine tegmental nuclei^84^. Additionally, hippocampal pyramidal neurons have been showed to be suppressed during *in vivo* recordings upon cerebral ventricle OXA infusions by indirect activation of local inhibitory interneurons^85^. In line with these studies, our data show that OXA application *in vivo* can have a suppressive outcome on spike firing. Because we did not observe hyperpolarisation of dLGN neurons during patch clamp recordings, we speculate that the suppressive effect may be due to activation of local GABAergic interneurons. In fact, interneurons in the dLGN make inhibitory connections with relay cells^86^. In our *in vivo* recordings, OXA-suppressed neurons constituted 7% and 14% of all neurons recorded during light and dark phase, respectively. Distinction regarding a neuronal type: thalamo-cortical vs local interneurons in *in vivo* approach was not made, as even though general criteria exist for such analysis, it has not been published before specifically for the rat dLGN cells. Patch clamp recordings would enabled such discrimination and experiment focusing on dLGN interneurons would help in interpretation of our data, however considering low percentage of interneurons in the dLGN (~10%), low response rate to OXA in the structure of adult rats and challenging methodological aspects regarding patch clamp recordings in adult animals, such experiment would not seem to draw any conclusions. Importantly, local OXA infusion was not limited to the recorded neuron; thus, in some cases, we may have also observed transsynaptically mediated effects.

OXA-responsive dLGN neurons could not be linked to any specific population of dLGN neurons. Some of them (40% and 60% recorded under the light and dark phase, respectively) were sensitive to light stimulation and were thus most likely directly engaged in vision formation. The majority were modulated by the general brain state according to the simultaneously recorded ECoG under urethane anaesthesia. Moreover, OXA-responsive neurons did not express the same firing pattern, as some of them were classified as infra-slow oscillatory during the light phase. Considering the large heterogeneity of dLGN neurons^86^, such a wide distribution of OXA-responsive neurons is not surprising. More importantly, these results extend those of Chrobok *et al*.^27^ by showing that OXA influences not only neurons contributing to direct vision formation but also dLGN neurons receiving signals from non-retinal inputs (for review see^86^). The very strong modulatory innervation of the dLGN derives from the brainstem cholinergic nuclei that control network states during arousal, sleep and wakefulness^87,88^. Interestingly, the present data show that some OXA-responsive dLGN neurons work in a state-dependent manner under urethane anaesthesia. These OXA-responsive neurons were more often found during the dark period, and they ‘preferred’ cortical activation than SWA state. Orexinergic neurons promote arousal and are more active during wakefulness in the dark period and become silent during sleep^18,19^. During naturally occurring slow-wave sleep, thalamo-cortical dLGN neurons are mostly hyperpolarized and generate lower firing rates, whereas during REM sleep and waking states, they are depolarised and fire more often^89,90^. Moreover, neurons switch between bursting and tonic firing modes, with the latter prevailing during slow-wave sleep^91–93^. These differences reflecting the state-dependence of dLGN neuron physiology have been examined in detail in terms of their light responsiveness^94–97^ and with little attention to the state-modulatory influences of pharmacological agent application^89,98^. Iontophoretical administration of acetylcholine to single dLGN cells caused excitation of firing only during waking and REM sleep^89^, whereas adrenergic receptor agonists worked mainly during slow-wave sleep^98^. On the other hand, OXA was showed to work in a state-dependent manner on the serotoninergic DRN neurons causing excitation only during naturally occurring sleep^75^. Our results coincide well with these studies, as we report that state-dependent OXA-responsive dLGN neurons responded during the cortical activation state and were more often observed during the dark period. These neurons were mostly modulated by the ECoG and were characterised by a higher firing rate during cortical activation than during SWA state. We hypothesise that state-dependent OXA-responsive dLGN neurons may be engaged in mediating arousal-related activity, however taking into consideration the heterogeneous nature of the dLGN and small number of such neurons; it may be difficult to test.

Three main conclusions can be driven from the present study. First, taking into consideration the relatively low number of OXA-responsive dLGN neurons recorded *in vivo* and *in vitro* in adult rats compared to previously published data on juvenile rats^27^ and sparse orexinergic innervation of the nucleus in both studies, we suggest that OXA is particularly important for the postnatal development of the visual thalamus. At present, however, its role in this process remains unclear and requires further investigation. Second, a comprehensive analysis of the electrophysiological properties of OXA-responsive dLGN neurons showed a large heterogeneity, thus extending its previously suggested role from modulation of visual processes to more global effects on dLGN functioning. OXA-responsive neurons were characterised as receiving not only visual information but also modulatory influences from the brainstem and/or cortex. Moreover, the state-dependent action of OXA, intensified during the dark period, further implicates its contribution to the mediation of circadian and arousal-related activity to the dLGN, which may result in the facilitation/attenuation of incoming information and/or information processed by the dLGN. Lastly, we emphasise the importance of circadian control of not only clock-related structures and simultaneous ECoG and spike recordings, especially in experiments on the thalamus.

## Methods

### Ethical approval

All experimental procedures were performed in accordance with Polish Animal Protection Law, approval (196/2012) of Local Ethics Committee of Jagiellonian University in Krakow and regulations and standards of the Directive 2010/63/EU of the European Parliament and of the Council of 22 September 2010 on the protection of animals used for scientific purposes, the 3Rs law, and the ARRIVE guidelines for reporting experiments involving living animals with respect to anaesthesia and animal handling^99^.

### Animals

All experiments, except for the immunohistochemistry study, were conducted on adult, male Wistar rats bred in the animal facility of the Institute of Zoology and Biomedical Research at Jagiellonian University in Krakow. Animals were housed under a 12:12-h light:dark cycle at a temperature of 22–23 °C and 60% humidity with food and water available *ad libitum*. Zeitgeber time (ZT) 0 was designated as the time of lights on. Experiments were performed during two different light regimes: light phase (ZT 1–11) and dark phase (ZT 13–23). Regardless of the experimental protocol (*in vivo* or *in vitro* study), animals were removed from their home cages during the early portion of the light (ZT 1–2) or dark (ZT 13–14) phase. For *in vivo* experiments, rats were directly anaesthetised with an i.p. injection of urethane (1.5 g/kg in 2 mL of 0.9% NaCl, Sigma-Aldrich, Poland). The dark phase group received the injection in a darkened room, and immediately after their eyes were covered with black foil. The surgery and electrophysiological recordings were performed in a darkened room with the light intensity at the animal’s eyes < 0.1 µW/cm^2^ through the experimental sessions. The rig was covered with a light-impermeable material. Electrophysiological experiments during the light phase were conducted in the same experimental room with room lights on (~100 µW/cm^2^). The electrophysiological recordings spanned the middle portion of the light (ZT 3–10) and dark (ZT 15–22) phases. The experimental design is shown in Fig. 1. A total of 64 rats were used for the study (47 for *in vivo*, 13 for *in vitro* and 4 for immunohistochemistry experiments).

### *In vivo* neurophysiology

#### Surgery and single-unit electrophysiological recordings

Adult Wistar rats weighing 270–400 g (2–3 months old) were used. Following urethane injection and prior to the surgery, each animal was checked for withdrawal and ocular reflexes to assess the level of anaesthesia (additionally supplemented with 10% of the initial dose of urethane, if necessary). The physiological state of the animal was continuously monitored by electrocardiogram and body temperature readouts. The animals’ head was secured in a stereotaxic apparatus (Advanced Stereotaxic Instruments, USA) and an initial incision was made to expose the scull surface. Skin and all soft tissues were removed to reveal sutures and stereotaxic points. A craniotomy was performed above the dLGN, 4–5 mm posterior and 3.3–4.3 mm lateral to *bregma*^100^. The dura was punctured, and the exposed brain tissue was covered with a drop of mineral oil (Sigma-Aldrich, Poland) to prevent drying. The recordings were taken at a depth of 4.0–5.5 mm from the first contact of the electrode with the cortical surface. Borosilicate glass micropipettes (resistance: 5–10 MΩ) pulled on a horizontal puller (P-97; Sutter Instrument Co., USA) and filled with 4% Chicago Sky Blue in 2% NaCl (ChSB; Sigma–Aldrich, Poland) were used to acquire neuronal signals. The recorded single-unit (sometimes multi-unit) activity was amplified 10,000 × , filtered between 300 Hz and 3 kHz and digitised at 20 kHz using an Axon Instruments CyberAmp 380 (Molecular Devices Corporation, USA), Micro mkII interface and Spike2 software (version: 7.10; Cambridge Electronic Design Inc., UK). Data were stored on a computer, and all further analyses were performed off-line.

### Local drug infusions

**Figure 7:**
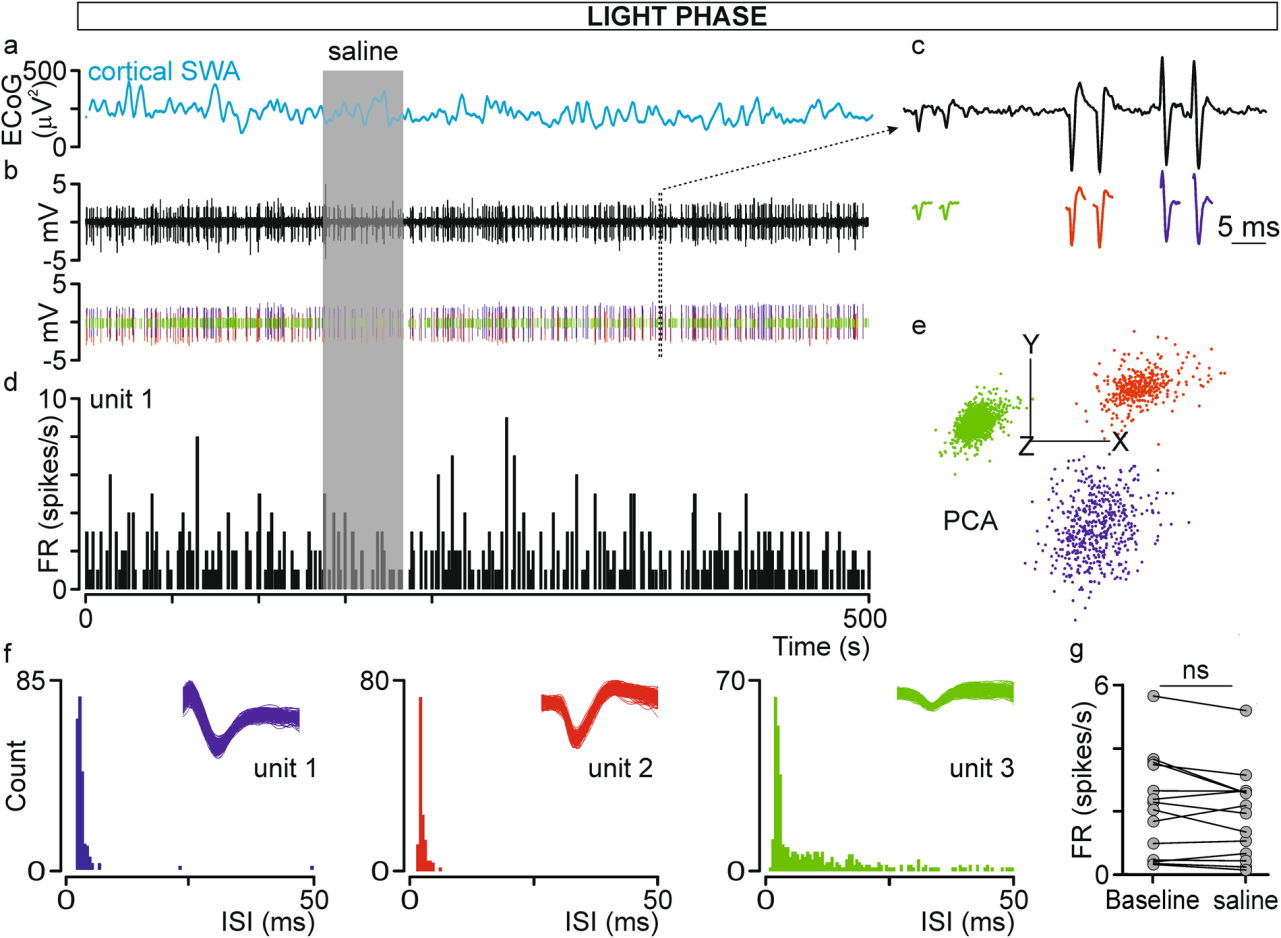
Control experiment involving saline pressure-driven infusion into the dLGN. (**a**) Delta-band ECoG power calculated from the raw ECoG trace and simultaneously acquired (**b**) raw neuronal signal from the dLGN. (**c**) The typical waveforms of extracellularly recorded dLGN neurons are visible on an expanded timescale, thereby allowing spike sorting (green, red and blue vertical lines below the raw trace in (**b**). (**d**) A firing rate histogram (bin size = 1 s) constructed for unite 1 (blue). (**e**) After spike extraction, waveforms were clustered and sorted into three units based on principal component analysis (PCA). (**f**) Distribution histograms of interspike intervals (ISIs) for all three sorted units confirming proper spike sorting. (**g**) The mean ± SEM firing rate for baseline and after saline infusion. Data were analysed by paired t-test, ns > 0.05.

The recording electrode was connected to a custom-made injection system enabling local, pressure-driven drug infusions (Fig. 1b). The injection system was built using a fused silica capillary tube (100 µm ID, 164 µm OD; Polymicro Technologies; www.molex.com, cat no. TSP100170) glued to the Tygon tube and connected to a 5-µL Hamilton syringe (Sigma-Aldrich, Poland). Next, the fused silica tube was glued 200–300 µm above the tip of the recording electrode under visual microscopic control. The same system was used 3 to 4 times and was tested for leaks and washed out with sterilized water each time. The system was pre-loaded with 200 µM OXA diluted in saline or saline and 200 nL of the fluid was infused. In control experiments (n = 14 neurons recorded during light phase and cortical SWA from 3 animals), 200 nL of saline was slowly infused during on-line monitoring cell activity to confirm outflow, signal stability and lack of cell response. There were no significant differences between the mean firing rate or CV before and after saline infusion (Fig. 7). The system was built based on Cerina *et al*.^101^.

### Electrocorticogram (ECoG) recordings

ECoG was simultaneously recorded with neuronal activity using a silver-wire electrode mounted on the stainless-steel screw contralateral to the recording site. The signal was amplified 1,000x and bandpass filtered between 0.5–30 Hz using a CyberAmp 380 amplifier equipped with a preamplifier (Axon Instruments, USA) and connected to CED Micro mkII interface and Spike2 software (version: 7.10; Cambridge Electronic Design Inc., UK).

### Light stimulations

The majority of recorded cells were tested for light responsiveness. Light stimulations were mostly presented during cortical activation phase (it is longer and more stable under urethane anaesthesia). Thus, the eye contralateral to the craniotomy (dLGN cells exhibited pure contralateral or binocular responses to light stimulations^102,103^) was held open, and the pupil was dilated by a topical application of atropine solution (Atropinum Sulfuricum WZF 1%, Polfa, Poland). The cornea was moisturized with mineral oil to prevent drying. A light-emitting diode (white, cold LED; 5 mm; OptoSupply Ltd., China; OSWWY25111E) was placed in front of the eye and connected to a Master-8 stimulator (A.M.P.I., Israel). Regardless of the experimental group (light or dark phase), all animals were presented with 5-s light pulses with 25-s intervals, repeated 6–8 times. The intensity of the light stimulus differed, as it was presented against photopic and scotopic backgrounds, and was 220 and 100 µW/cm^2^ for the light and dark group, respectively. The light intensity was measured with a GENTEC P-LINK (Gentec Electro-Optics, Canada).

### Histological verifications

To mark the recording sites, at the end of each experiment, an anodal current (20 μA) was ejected for 15 min from the recording electrode containing ChSB (Fig. 2). Next, rats were overdosed with sodium-pentobarbital (Biovet, Poland) and subsequently intracardially perfused with 0.1 M phosphate-buffered saline (PBS) and 4% paraformaldehyde (PFA) in PBS (pH = 7.4). Brains were removed and post-fixed in PFA for ~48 h. Coronal slices (100 μm thick) were cut on a vibroslicer (Leica VT1000S, Germany), and all sequential sections containing the dLGN were collected. ChSB marks were verified with a light microscope and mapped onto corresponding coronal schemes of the stereotactic atlas of the rat brain^100^.

### Data analysis

Waveforms extracted from the continuous signal were sorted off-line using the template matching method and principal component analysis (PCA; Fig. 7) in Spike2 software (version: 7.10; Cambridge Electronic Design, Inc., UK). Moreover, proper spike sorting was confirmed by interspike interval (ISI) histograms.

Simultaneously recorded alternating changes in ECoG signal: cortical SWA and activation states were identified based on the work of Clement *et al*.^69^ and were characterised by a dominant frequency, delta (~1 Hz) and theta (~4 Hz), respectively.

The relationship between the firing rate of a single dLGN neuron and ECoG changes was established as in^31^. Briefly, a cross-correlation coefficient (CC) analysis was performed on delta power band derived from ECoG signals containing both activated and SWA phases (at least one alteration in ECoG) and simultaneously recorded FR of the dLGN neurons. Neurons were characterised as significantly correlated with the ECoG when their maximal CC values exceeded confidence limits representing a 2-fold increase in their respective standard errors. Based on maximal CC values, dLGN neurons were classified into SWA-ON and activation-ON (Supplementary Fig. S1 and Table S1). Cross-correlation analysis was performed using Statistica software (StatSoft, Inc., USA).

dLGN neurons were classified as light responsive based on the peristimulus time histograms (PSTHs, bin size = 0.1 s, Supplementary Fig. S1) calculated in Spike 2 software (version: 7.10; Cambridge Electronic Design, Inc., UK).

The regularity of firing was assessed based on the coefficient of variation (CV) calculated as a ratio of the SD of the ISI to its mean. The Young’s regularity criterion was used^104^, and neurons in which CVs exceeded 0.35 were classified as non-regular.

The infra-slow oscillatory activity was identified by applying fast Fourier transform and autocorrelation analysis to ~900-s-long intervals, as shown before in detail^105^.

To characterise dLGN neurons as OXA-responsive, we used a similar method as Chen *et al*.^62^. The baseline firing rate was averaged for at least 50 s (max 200 s when examining infra-slow oscillatory pattern of activity) before OXA application with the condition that the recording had to be obtained under one cortical phase. To assess the beginning and end of the response to OXA, the firing rate was averaged for 10-s bins with a 1-s sliding window starting from 10 s before OXA infusion (the averaged firing rate of the first second starting from the beginning of infusion was calculated as the averaged firing rate of 9 s before and the 1 s from the beginning of the infusion). An increase or decrease in firing rate was considered significant if it exceeded or fell below 3 standard deviations (SDs). The SD was calculated by averaging the firing rate of sequential time bins (10 s) within 50 s before OXA application.

Statistical comparisons were based on the results of two-way ANOVA followed by Tukey’s test, paired/unpaired t tests, Mann–Whitney, Kruskal–Wallis, Wilcoxon tests, Fisher’s and Chi-square exact tests performed in Prism, version 5 (GraphPad Software Inc., USA). Significance was set at p < 0.05. Data are presented as the mean ± SEM.

### Patch clamp recordings

#### Tissue

To obtain brain slices containing the dLGN, we used a previously described procedure^59^. In short, adult male Wistar rats (5–6 weeks old) were anaesthetised with isoflurane (2 ml/kg body weight; Baxter, Poland) and decapitated between ZT 1–2 or ZT 13–14. Next, the brain was quickly removed and immersed in ice-cold, oxygenated (carbogen, 95% O_2_, 5% CO_2_) cutting artificial cerebrospinal fluid (ACSF), composed of (in mM) 185 sucrose, 25 NaHCO_3_, 3 KCl, 1.2 NaH_2_PO_4_, 2 CaCl_2_, 10 MgCl_2_ and 10 glucose. Then, the block of brain tissue containing the thalamus was placed on the cold plate of the vibroslicer (Leica VT1000S, Germany). Coronal slices, 250 µm in thickness, including the dLGN, were cut and transferred to the pre-incubation chamber filled with recording ACSF containing (in mM) 123 NaCl, 25 NaHCO_3_, 3 KCl, 1.2 NaH_2_PO_4_, 2 CaCl_2_, 2 MgCl_2_ and 10 glucose. Slices were kept in this solution for 30 min at 32 °C, followed by 60 min at room temperature before placement in the recording chamber (21 °C–24 °C).

### Whole-cell recordings

Recording electrodes were placed in the dLGN under visual microscopic control (40× objective of a Zeiss Examiner microscope fitted with infrared differential interference contrast (Germany). Recordings were performed in whole-cell configuration obtained by applying negative pressure from an Ez-gSEAL100B Pressure Controller (Neo Biosystem, USA). The recorded signal was amplified by a SC 05LX amplifier (NPI, Germany), low-pass filtered at 3 kHz and digitized at 20 kHz. Spike2 and Signal (Cambridge Electronic Design, Inc., UK) software were used for the recordings. A liquid junction potential of approximately −13 mV was added to the measured membrane potential. Typical recordings lasted ~1000 s.

Experiments in current clamp mode (holding current = 0 pA) were conducted at room temperature (21 °C–24 °C). Patch pipettes pulled on a horizontal puller (P-97; Sutter Instrument Co., USA) were filled with normal intrapipette solution containing (in mM) 125 potassium gluconate, 20 KCl, 10 HEPES, 2 MgCl_2_, 4 Na_2_ATP, 0.4 Na_3_GTP, 1 EGTA and 0.05% biocytin (pH = 7.4 adjusted with 5 M KOH; osmolarity ~ 300 mOsmol/kg). Rectangular current pulses (1 s, 80 pA) were applied to monitor the membrane resistance during each recording. Neurons for which the membrane resistance changed by more than 15% during the recording or that had a membrane potential more positive than −35 mV (−50 mV when adjusted for the junction potential) were excluded from further analysis.

### Data analysis

All data were analysed using MATLAB (MathWorks, Inc., USA) and GraphPad Prism 6 (GraphPad Software, Inc., USA) software. Changes in the firing rate and membrane potential were considered to be significant if they differed from baseline by more than three standard deviations (SDs). Data are presented as the mean ± SEM or maximal change of membrane potential ± SEM (Δ_max_). Δ_max_ is described as the highest point of the examined value reached during the response.

### Drugs

OXA (Tocris, Bristol, UK) was freshly prepared from 200 µM stocks (dissolved in saline), and 200 µM or 200 nM was used for the *in vivo* or *in vitro* study, respectively.

### Immunohistochemistry - OXA fibres

Four male juvenile Wistar rats (14–21 days old) were deeply anaesthetised with pentobarbital (100 mg/kg, i.p.) and sacrificed (ZT 1–2) by transcardial perfusion with PBS followed by 4% PFA. Brains were quickly removed and immersed in 4% PFA overnight. Next, blocks of tissue containing the dLGN were cut into 100- µm-thick slices on a vibroslicer (Leica VT1000S, Germany) and rinsed twice in PBS. Then, slices were transferred to solution containing 0.6% Triton X-100 (Sigma-Aldrich, Poland) and 10% NDS (Jackson ImmunoResearch Europe Ltd., UK) at room temperature. After 2 h, slices were rinsed in fresh PBS and incubated with goat OXA antisera (1:200, Santa Cruz Biotechnology, USA) in PBS, 2% NDS and 0.3% Triton X-100 overnight at 4 °C. Next, slices were washed twice in PBS and incubated with secondary anti-goat AlexaFluor 488-conjugated antibodies (1:300; Jackson ImmunoResearch Europe Ltd., UK) for 24 h. Finally, slices were rinsed in PBS and mounted on glass slides in Fluoroshield^TM^ (Sigma-Aldrich, Poland). Slices were then scanned on an A1-Si Nikon Inc. (Japan) confocal laser scanning system built on an inverted Nikon Ti-E microscope (Japan). Image processing was performed with the use of ImageJ2 software (version: 2.0.0-rc41/1.50d, NIH, USA). Quantitative analysis of the orexinergic fibres was performed using similar protocol to Chrobok *et al*.^27^ and the whole area of the dLGN was scanned. The results are presented as area fraction (%) ± SEM (total sum of immunoreactive pixels divided by the area of the dLGN).

## Supplementary information

Supplementary Information

## Data Availability

The datasets generated and analysed during the current study are available from the corresponding authors on reasonable request.
