# Supplementary material for: Orexin A as a modulator of dorsal lateral geniculate neuronal activity: a comprehensive electrophysiological study on adult rats
Source: Sci Rep. 2019 Nov 13;9:16729. doi: 10.1038/s41598-019-53012-9 (PMC6853907; doi:10.1038/s41598-019-53012-9)
Supplement: Supplementary file 1 — Supplementary Information [file 41598_2019_53012_MOESM1_ESM.pdf]

Orexin A as a modulator of dorsal lateral geniculate neuronal activity: a comprehensive electrophysiological study on adult rats

Patrycja Orlowska-Feuer[1,2,*], Magdalena Kinga Smyk[1,2], Katarzyna Palus-Chramiec[2], Katarzyna Dyl[2], Marian Henryk Lewandowski[2,*]

Affiliations:

[1] Malopolska Centre of Biotechnology (MCB), Jagiellonian University in Krakow, Krakow, Poland

[2] Department of Neurophysiology and Chronobiology, Institute of Zoology and Biomedical Research, Jagiellonian University in Krakow, Krakow, Poland

Supplementary Table S1: Electrophysiological properties of dLGN neurons recorded under light and dark phase. The data are presented as mean ± SD.

<table>
<tr><th>Electrophysiological property</th><th colspan="2">Light Phase</th><th colspan="2">Dark Phase</th><th>Comparison</th></tr>
<tr><td>No. of recorded cells</td><td colspan="2">118</td><td colspan="2">117</td><td></td></tr>
<tr><td>No. of light responsive neurons</td><td colspan="2">49 out of 111 tested neurons</td><td colspan="2">78 out of 117 tested neurons</td><td>Fisher’s exact test, p = 0.0008</td></tr>
<tr><td>Types of light responses (assessed during SWA phase)</td><td colspan="2">transient ON: 22, OFF: 14, sustained: 5</td><td colspan="2">transient ON: 25, OFF: 13, sustained: 21</td><td></td></tr>
<tr><td>Correlation between FR and ECoG[1]</td><td colspan="2">69 out of 78 tested neurons
CC = -0.35 ± 0.05</td><td colspan="2">70 out of 75 tested neurons
CC = -0.37 ± 0.04</td><td>Fisher’s exact test, p = 0.4026
CC: Mann-Whitney test, Two-tailed, P = 0.7295, U = 1753</td></tr>
<tr><td>Activation-ON neurons (characterised by negative CC values)</td><td colspan="2">54</td><td colspan="2">59</td><td rowspan="3">Chi-square test, p = 0.3826</td></tr>
<tr><td>SWA-ON(characterised by positive CC values)</td><td colspan="2">15</td><td colspan="2">11</td></tr>
<tr><td>Not correlated</td><td colspan="2">9</td><td colspan="2">5</td></tr>
<tr><td>FR SWA-ON cells</td><td>Cortical SWA</td><td>Cortical activation</td><td>Cortical SWA</td><td>Cortical activation</td><td>two-way ANOVA,</td></tr>
</table>

	3.58 ± 2.38 Hz	6.47 ± 6.97 Hz	4.34 ± 2.37 Hz	7.01 ± 6.27 Hz	interaction: p = 0.942, cortical state: p <0.0001, light phase: p = 0.0563
**FR activation-ON**	Cortical SWA	Cortical activation	Cortical SWA	Cortical activation	two-way ANOVA, interaction: p = 0.1820, cortical state: p = 0.2244, light phase: p = 0.0009
	5.56 ± 6.37 Hz	11.55 ± 9.13 Hz	3.70 ± 4.19 Hz	9.83 ± 7.59 Hz	
**FR not correlated**	Cortical SWA	Cortical activation	Cortical SWA	Cortical activation	two-way ANOVA, interaction: p = 0.2152, cortical state: p = 0.1997, light phase: p = 0.0850
	2.63 ± 1.47 Hz	2.72 ± 1.56	3.62 ± 5.68 Hz	8.49 ± 9.74 Hz	
**CV SWA-ON cells**	Cortical SWA	Cortical activation	Cortical SWA	Cortical activation	two-way ANOVA, interaction: p = 0.5205, cortical phase factor: p = 0.0920, light phase factor: p = 0.1183
	1.20 ± 0.29	1.14 ± 0.17	1.15 ± 0.19	1.01 ± 0.11	
**CV activation-ON cells**	Cortical SWA	Cortical activation	Cortical SWA	Cortical activation	two-way ANOVA, interaction: p = 0.8313, cortical phase factor: p = 0.0002, light phase factor: p = 0.1839
	1.26 ± 0.41	1.06 ± 0.20	1.18 ± 0.56	1.01 ± 0.16	
**CV not correlated**	Cortical SWA	Cortical activation	Cortical SWA	Cortical activation	two-way ANOVA, interaction: p = 0.0053, cortical phase factor: p = 0.015, light phase factor: p = 0.2068
	1.17 ± 0.14	1.13 ± 0.20	1.44 ± 0.14	1.02 ± 0.11	
**Resting membrane potential**	-70.44 ± 3.30 mV		-68.20 ± 4.8 mV		Unpaired t test, p=0.034
**Membrane resistance**	267 ± 56 MΩ		340 ± 100 MΩ		Mann-Whitney test, p = 0.011

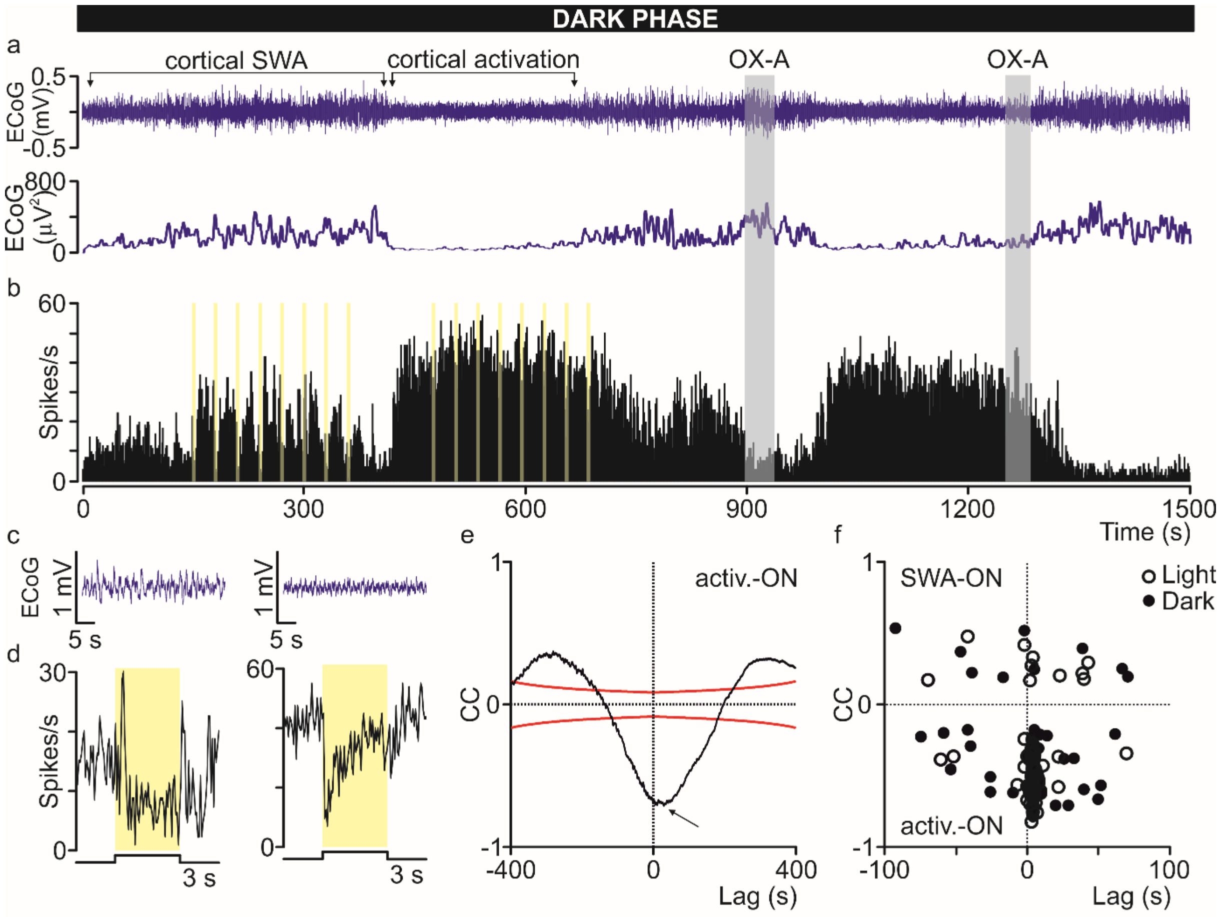

FIG. S1

Supplementary Fig. S1 Cortical state alterations influence dLGN neuron activity. Representative simultaneous recordings of a) ECoG (upper panel – raw trace, bottom panel – changes in delta-band ECoG) and b) firing rate of the dLGN neuron (bin size = 1 s) recorded during the dark phase. The grey rectangles correspond to the time of OXA pressure infusions. The yellow lines represent the time of light stimulation during c) cortical SWA and activation states, respectively. d) Adequate PSTHs (bin size = 0.01 s) confirming the neuron's sensitivity to 5-s light pulses (100 µW/cm^2) during cortical SWA and activation states but with different types of response: transient ON and OFF[1,2], respectively. e) Cross-correlation coefficient (CC) analysis showed that presented dLGN neuron was significantly correlated with delta activity in the ECoG and was characterised as activation-ON neuron (maximum peak in negative CC values). f) Scatter plot showing the distribution of CC values and lags across all recorded neurons under light (white circles) and dark (black circles) phases.

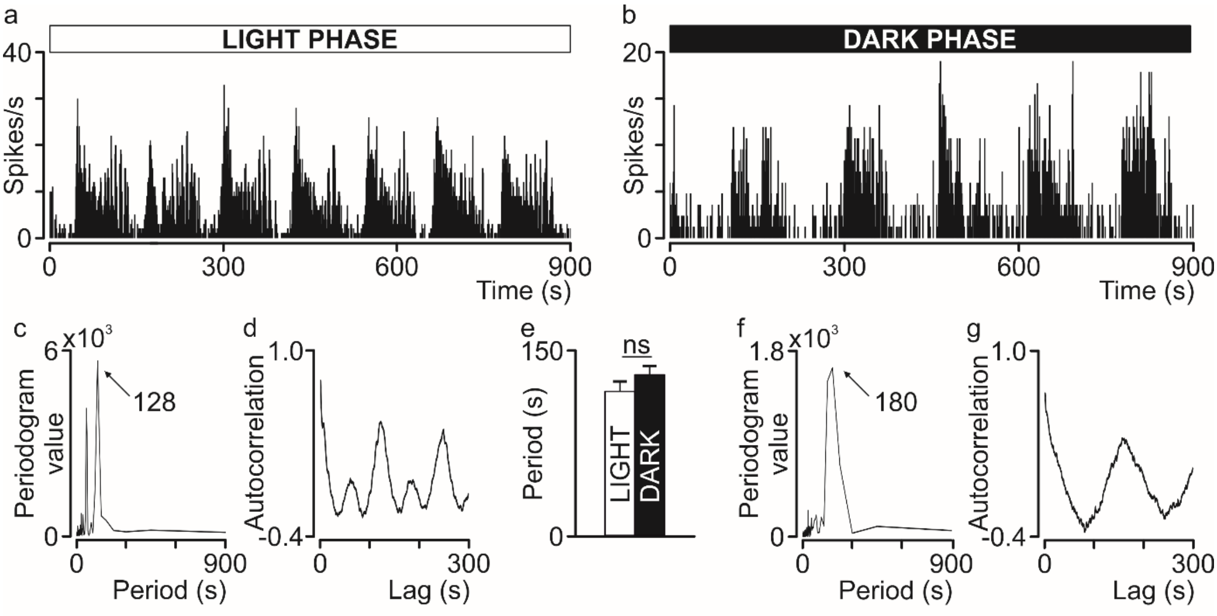

FIG.S2

Supplementary Fig. S2:Infra-slow oscillatory activity in the rat dLGN across the light-dark cycle. a-b) Firing rate histograms (bin size = 1 s) for representative infra-slow oscillatory neurons recorded under a) light and b) dark phases. c, f) FFT and d, g) autocorrelation analysis was performed for the recordings shown in a) and b), respectively, to characterise cells as oscillatory. e) The mean period ± SEM did not differ between light regimes. Data were analysed by the Mann-Whitney test, ns > 0.05.

REFERENCES

1. Jeczmien-Lazur, J. S., Orlowska-Feuer, P., Smyk, M. K. & Lewandowski, M. H. Modulation of spontaneous and light-induced activity in the rat dorsal lateral geniculate nucleus by general brain state alterations under urethane anaesthesia. *Neuroscience* **413**, 279-293 (2019).
2. Pietersen, A. N. J. *et al.* Relationship between cortical state and spiking activity in the lateral geniculate nucleus of marmosets. *J. Physiol.* **595**, 4475–4492 (2017).
